# Effects of medical service fee revision on reducing irrational psychotropic polypharmacy in Japan: an interrupted time-series analysis

**DOI:** 10.1007/s00127-021-02147-0

**Published:** 2021-07-31

**Authors:** Yusuke Okada, Manabu Akazawa

**Affiliations:** Department of Public Health and Epidemiology, Meiji Pharmaceutical University, 2-522-1, Noshio, Kiyose, Tokyo 204-8588 Japan

**Keywords:** Polypharmacy, Anxiolytics, Hypnotics, Antipsychotics, Antidepressants

## Abstract

**Purpose:**

According to the revised Japanese medical service fees aimed at reducing irrational psychotropic polypharmacy, medical service fees are reduced if the number of simultaneously prescribed psychotropic drugs exceeds the standard. This study primarily aims to examine the effect of the 2018 revision.

**Methods:**

Using a large Japanese administrative claims database, we retrospectively identified five groups (April 2013–September 2018) prescribed at least one drug from the following drug groups: anxiolytics, hypnotics, sum of anxiolytics and hypnotics, antipsychotics, and antidepressants (study population in each group: 547,511, 406,524, 759,137, 112,929, and 201,046, respectively). We used an interrupted time-series design to evaluate changes in the proportion of patients prescribed more than the standard number of drugs.

**Results:**

After the 2018 revision, the proportion of patients prescribed more than the standard number of drugs significantly decreased only for the sum of anxiolytics and hypnotics; estimated changes in level and trend were − 0.60% [− 0.69%, − 0.52%] and − 0.04% [− 0.06%, − 0.02%] per month, respectively. The proportion of patients exhibiting a decrease in the number of prescribed drugs from more than the standard to within the standard increased when the revision was enforced (April 2018); this proportion in April 2018 was 36.3%, while all other proportions were in the range of 12.1–22.3%.

**Conclusion:**

The 2018 revision promoted a reduction in the number of prescribed drugs, which served as an important factor in the decrease in the proportion of patients prescribed more than the standard number of drugs for the sum of anxiolytics and hypnotics.

**Supplementary Information:**

The online version contains supplementary material available at 10.1007/s00127-021-02147-0.

## Introduction

Psychotropic polypharmacy usually refers to combination therapy with two or more psychotropic drugs [1]. Psychotropic polypharmacy is inherently associated with an increased risk of adverse events or drug–drug interactions and with adherence issues, because the treatment regimen becomes more complicated [1–3]. Although psychotropic polypharmacy is appropriate in certain situations, irrational psychotropic polypharmacy such as combination of drugs with highly similar mechanisms should be avoided [1]. In addition, several clinical guidelines for the treatment of schizophrenia or major depressive disorders, including guidelines developed by Japanese societies, generally recommend monotherapy [4–11]. However, some patients are treated with psychotropic polypharmacy, including irrational polypharmacy; the most frequent reason for psychotropic polypharmacy is that a single medication does not achieve a treatment goal [1].

In Japan, the Ministry of Health, Labour and Welfare (MHLW) made four revisions to medical service fees aimed at reducing irrational psychotropic polypharmacy [12]. Medical service fees are the fees received by medical institutions and pharmacies serving insured persons, as the price of insured medical services [13]. All healthcare providers throughout Japan are required to comply with the medical service fees set by the MHLW, and providers are prohibited from charging fees in excess of these set amounts. The main point of these four revisions was that medical service fees (such as prescription fee and drug fee) were reduced if the number of simultaneously prescribed psychotropic drugs exceeded the standards set by the MHLW; nevertheless, there were some exceptions. In addition, concomitant prescription of more than the standard number of drugs are not based on evidence; therefore, each revision was expected to reduce irrational or unnecessary psychotropic polypharmacy. The first revision was enforced in April 2012. According to the 2012 revision, the fee for psychiatric outpatient services/consultation was reduced if three or more anxiolytics or three or more hypnotics were prescribed simultaneously; the standard number of prescribed drugs was two. According to the second revision enforced in October 2014 (notified in April 2014), prescription fee and drug fee were reduced if three or more anxiolytics, three or more hypnotics, four or more antipsychotics, or four or more antidepressants were prescribed simultaneously; the standard number of prescribed drugs were two for anxiolytics and hypnotics and three for antipsychotics and antidepressants. In the third revision, enforced in April 2016, the standard number of prescribed antipsychotics and antidepressants changed from three to two. In the fourth revision, enforced in April 2018, the prescription fee and drug fee were reduced if the total number of anxiolytics and hypnotics simultaneously prescribed was four or more; the standard number was three. In addition, the prescription fee was reduced if anxiolytics or hypnotics were prescribed for more than 12 months at the same dosage and administration. The revision was introduced mainly because benzodiazepine receptor agonists are a major group of anxiolytics and hypnotics that pose a risk of tolerance, dependence, and other side effects [14, 15].

Several studies have investigated the effect of these revisions. Okumura et al. investigated the effect of the revisions in 2012 and 2014 using an out-of-hospital prescription database. They reported that the proportion of prescriptions containing three or more anxiolytics decreased from 1.6% in April 2011 to 0.9% in November 2014, and the proportion of prescriptions with three or more hypnotics decreased from 4.5% to 2.4% [16]. Hirano et al. investigated the effect of the revisions in 2012, 2014, and 2016 using an administrative claims database. They reported that the proportion of patients prescribed three or more anxiolytics and the proportion of patients prescribed three or more hypnotics decreased after the notification and enforcement of the revision in 2014. In addition, they reported that the proportion of patients prescribed three or more antipsychotics and the proportion of patients prescribed three or more antidepressants decreased after the revision in 2016 [17].

However, the effect of the revision in 2018 has not been investigated. In addition, the effect on prescription changes at an individual patient level is unclear, because there are two possible reasons for the decrease in the proportion of patients prescribed more than the standard number of drugs: (a) healthcare providers more frequently reduced the number of prescribed drugs for patients already prescribed more than the standard number of drugs than before or (b) healthcare providers less frequently started to prescribe more than the standard number of drugs for patients prescribed drugs within the standard number than before. The latter indirectly contributes to a relative increase in the frequency of reducing the number of prescribed drugs. The major reason behind the difficulty of reducing psychotropic drugs is that withdrawal or dose reduction of psychotropic drugs may lead to relapse or recurrence of the disease being treated or withdrawal syndromes [18–23]. In addition, several guidelines do not specify when and how to reduce drugs for patients who are already prescribed more than the standard number of drugs, especially for long-time users [4–9, 24–26]. Thus, owing to the difficulty in reducing the prescribed number of psychotropic drugs, a decrease in the proportion of patients prescribed more than the standard number of drugs might essentially be, because healthcare providers avoided starting to prescribe more than the standard number of drugs.

Our study, therefore, aims to examine the effect of the revision in 2018 on the proportion of patients prescribed more than the standard number of drugs. In addition, our study aims to examine the effect of the revisions in 2014, 2016, and 2018 on the changes in drug prescription at an individual patient level.

## Methods

### Data source

We used a commercially available administrative claims database maintained by JMDC Co. Ltd (Tokyo, Japan) [27]. The JMDC claims database includes medical claims, diagnosis procedure combination claims, and pharmacy claims data from beneficiaries insured by health insurance societies. The number of beneficiaries was approximately 3.8 million in September 2018. Beneficiaries in the database are employed workers and their families; thus, data for patients aged 65 or more are limited. In addition, the database does not include data from patients aged 75 or older, because they are insured by another health insurance called long life medical care system (medical care system for elderly in the latter stage of life) [28], which the database does not cover.

### Study population

We defined five groups in the study population based on the claims data from April 2013 to September 2018 as follows: (i) patients who were prescribed at least one anxiolytic, (ii) patients who were prescribed at least one hypnotic, (iii) patients who were prescribed at least one antipsychotic, (iv) patients who were prescribed at least one antidepressant, and (v) patients who were prescribed at least one anxiolytic or hypnotic. We allowed that a patient of each group belong to other groups, because our study evaluates longitudinal changes of outcome within each group. For counting the number of prescribed drugs, we defined each drug category on the basis of active ingredients listed by the MHLW [Supplemental Table S1]. However, injectable antipsychotics, except for long-acting injectable antipsychotics, were excluded in this study even if the listed active ingredients were used, because these drugs were used only temporarily; including these drugs for counting would lead to overestimation of the proportion of patients who were continuously prescribed more than the standard number of drugs. In addition, other non-oral psychotropic drugs such as barbituric acid injectable hypnotics or benzodiazepine suppository drugs were excluded, because these drugs are often used for purposes different from that of anxiolytics or hypnotics.

### Outcome measures

The outcome for evaluating the effect of the revision at the group level was the proportion of patients who were prescribed more than the standard number of drugs in each month. The denominator was the number of patients who were prescribed at least one drug for each drug category. The numerator was the number of patients who were prescribed more than the standard number of drugs; the standard number was two for anxiolytics, hypnotics, antipsychotics, and antidepressants, and three for the sum of anxiolytics and hypnotics. We determined the number of prescribed drugs based on active ingredient names only in the same medical facility and on the same day. We subsequently used the largest number of drugs as the number of prescribed drugs in each month.

**Fig. 1:**
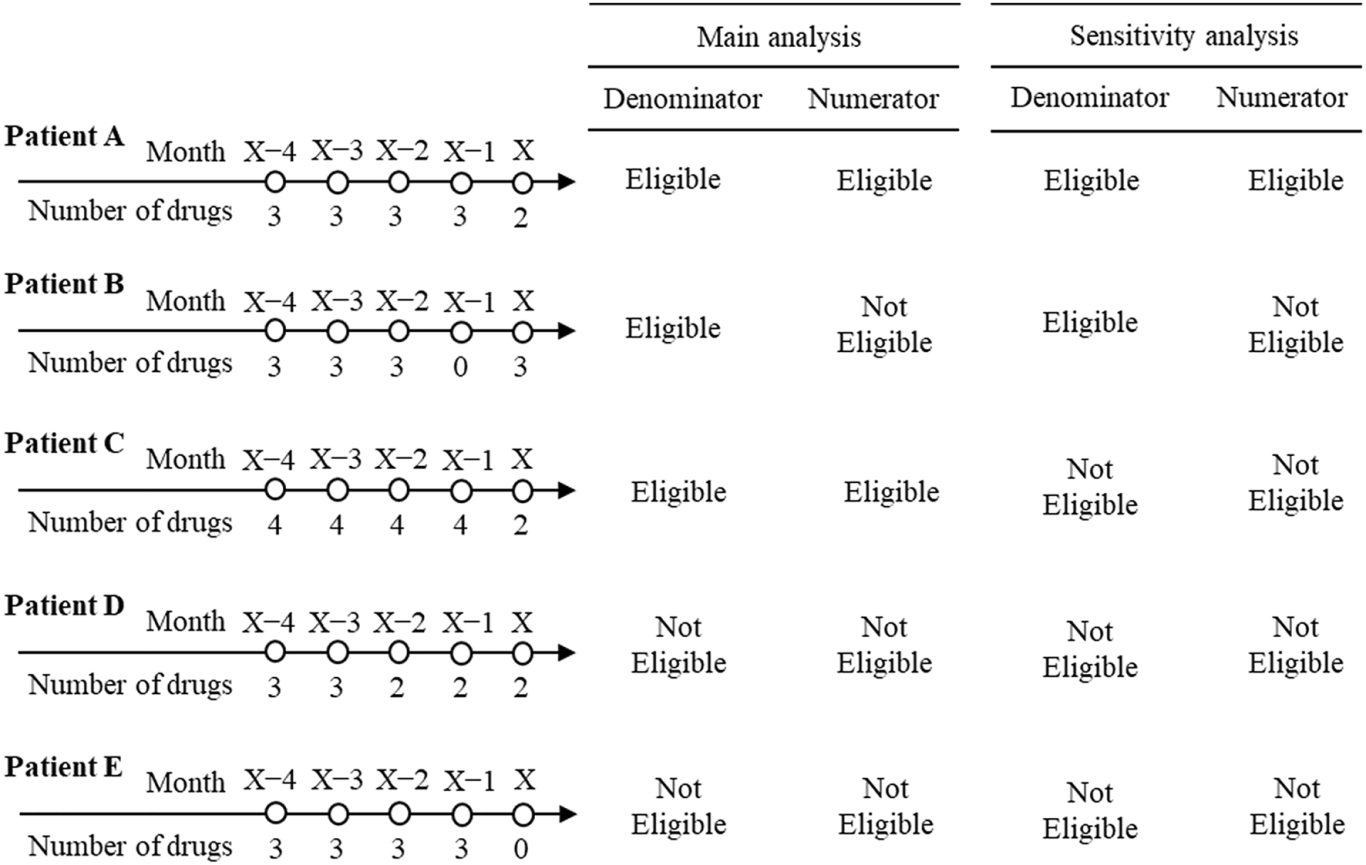
Examples of eligibility of denominator and numerator in month *X* for main analysis and sensitivity analysis. The standard number is two in these examples. Patient B is not eligible for numerator of main analysis and sensitivity analysis, because the number of drugs in month *X* is over the standard number. Patient C is not eligible for denominator and numerator of sensitivity analysis, because the number of drugs in month *X* − 1 is over the standard number plus one. Patient D is not eligible, because the number of drugs in month *X* − 1, the most recent month with at least one drug prescription from *X* − 4 to *X* − 1 in this case, is not over the standard number. Patient E is not eligible, because the number of drugs in month *X* is zero

Outcomes for evaluating the effect of the revisions at an individual level were the proportion of patients exhibiting a decrease in the number of prescribed drugs from more than the standard number to within the standard number in each month. The denominator of month *X* for each drug category was the number of patients who met the following conditions: (i) at least one drug was prescribed in month *X* (assuming that healthcare providers prescribe at least one drug for patients who were prescribed more than the standard number of drugs, when trying to reduce drugs), (ii) the number of prescribed drugs in the most recent month with at least one drug prescription from *X* − 4 to *X* − 1 was more than the standard number. The numerator of month *X* was the number of patients who were eligible for the denominator and prescribed drugs within the standard number in month *X*. Since it is difficult to understand longitudinal changes in the outcome if the proportion of patients prescribed only the standard number plus one of drugs extremely vary relative to patients prescribed more than the standard number of drugs in each month. Thus, sensitivity analysis was conducted as follows: we changed the second condition of the denominator to “the number of prescribed drugs in the most recent month with at least one drug prescription from *X* − 4 to *X* − 1 was only the standard number plus one.” Examples of the eligibility of the denominator and numerator for the main analysis and sensitivity analysis are shown in Fig. 1. Only for the sum of anxiolytics and hypnotics, we defined three subgroups based on the type of drugs that decreased in the following order: only anxiolytics, only hypnotics, and both anxiolytics and hypnotics. These subgroups were used only for descriptive analysis (not for the joinpoint regression analysis).

### Statistical analysis

We performed a segmented regression analysis using an interrupted time-series design to estimate changes in levels and trends of the proportion of patients prescribed more than the standard number of drugs [29]. We defined the five segments as follows: the period before the revision in 2014 (from April 2013 to March 2014), the period after notification of the revision in 2014 (from April 2014 to September 2014), the period after enforcement of the revision in 2014 (from October 2014 to March 2016), the period after the revision in 2016 (from April 2016 to March 2018), and the period after the revision in 2018 (from April 2018 to September 2018). Each segmented linear regression model included terms to estimate the baseline level (intercept), baseline trend, changes in level immediately after each revision, changes in trend after each revision, and error term. Each model was controlled for autocorrelation using the most parsimonious autoregressive error model, which was identified using backward elimination. We evaluated the adequacy of the models on the basis of residual analyses and Durbin–Watson statistics. Values of *p* < 0.05 (two-sided) were considered statistically significant. Analyses were conducted using the SAS software, version 9.4.

We used a joinpoint regression analysis, which is useful for identifying and describing changes in trend data [30, 31], to estimate changes in the proportions of patients exhibiting a decrease in the number of prescribed drugs. The joinpoint regression model consists of multiple continuous linear segments connected at the change points (joinpoints), and it does not require the pre-defined joinpoints. We chose the grid search method. The Monte Carlo permutation test was used for model selection for determining the best-fitting model, allowing a maximum of five joinpoints and a minimum of 2 months between joinpoints. The overall significance level was set at 0.05. We considered that there were no significant changes if no joinpoints were identified. Analyses were conducted using the Joinpoint Regression Program (Version 4.8.0.1 April 2020; Statistical Research and Applications Branch, National Cancer Institute.)

## Results

### Patient characteristics

The total number of patients who were prescribed at least one drug from April 2013 to September 2018 were 547,511 (anxiolytics), 406,524 (hypnotics), 759,137 (sum of anxiolytics and hypnotics), 112,929 (antipsychotics), and 201,046 (antidepressants). Table 1 lists the characteristics of patients who were prescribed at least one drug from each drug category and each segment. No remarkable changes regarding age and sex were observed throughout the five segments for all drug categories. The proportion (%) of patients prescribed psychotropic drugs of other categories in the initial month of prescription records were higher in segment 2 and segment 5 than other segments for anxiolytics, hypnotics, and sum of anxiolytics and hypnotics.

**Table 1:** Patient characteristics

Drug category	Characteristic	Segment 1	Segment 2	Segment 3	Segment 4	Segment 5
		Apr 2013–Mar 2014	Apr 2014–Sep 2014	Oct 2014–Mar 2016	Apr 2016–Mar 2018	Apr 2018–Sep 2018
Anxiolytics	Patiens, *n*	149,166	107,192	236,661	284,471	125,853
	Age, years					
	Mean (SD)^a^	42.3 (16.1)	43.4 (15.4)	43.1 (15.8)	42.9 (15.8)	44.9 (14.8)
	≤ 19 years, *n* (%)^a^	13,904 (9.3)	8124 (7.6)	19,836 (8.4)	24,392 (8.6)	7881 (6.3)
	≥ 65 years, *n* (%)^a^	8970 (6.0)	7035 (6.6)	14,770 (6.3)	6764 (5.9)	6990 (6.6)
	Male, *n* (%)	71,548 (48.0)	51,439 (48.0)	113,144 (47.8)	136,993 (48.2)	61,784 (49.1)
	Concomitant drugs					
	Hypnotics, *n* (%)^b^	35,469 (23.8)	28,337 (26.4)	55,589 (23.5)	66,816 (23.5)	36,429 (28.9)
	Antipsychotics, *n* (%)^b^	11,109 (7.4)	9731 (9.1)	18,112 (7.7)	21,639 (7.6)	13,256 (10.5)
	Antidepressants, *n* (%)^b^	32,132 (21.5)	27,446 (25.6)	49,180 (20.8)	57,414 (20.2)	35,324 (28.1)
Hypnotics	Patiens, *n*	108,099	82,809	179,667	226,849	114,832
	Age, years					
	Mean (SD) ^a^	43.3 (17.5)	44.6 (16.7)	43.8 (17.2)	43.5 (17.2)	45.8 (15.9)
	≤ 19 years, *n* (%)^a^	10,944 (10.1)	6789 (8.2)	17,169 (9.6)	22,314 (9.8)	8260 (7.2)
	≥ 65 years, *n* (%)^a^	9145 (8.5)	7542 (9.1)	14,982 (8.4)	17,332 (7.8)	9588 (8.3)
	Male, *n* (%)	57,216 (52.9)	44,288 (53.5)	95,553 (53.2)	121,330 (53.5)	62,485 (54.4)
	Concomitant drugs					
	Anxiolytics, *n* (%)^b^	36,726 (34.0)	29,133 (35.2)	57,104 (31.8)	68,748 (30.3)	36,842 (32.1)
	Antipsychotics, *n* (%)^b^	12,688 (11.7)	11,413 (13.8)	20,764 (11.6)	25,395 (11.2)	16,998 (14.8)
	Antidepressants, *n* (%)^b^	26,139 (24.2)	22,779 (27.5)	41,549 (23.1)	50,427 (22.2)	32,775 (28.5)
Sum of anxiolytics and hypnotics	Patiens, *n*	211,023	156,369	340,052	414,253	197,854
	Age, years					
	Mean (SD) ^a^	42.3 (17.2)	43.7 (16.5)	43.0 (16.9)	42.8 (16.9)	45.1 (15.8)
	≤ 19 years, *n* (%)^a^	22,848 (10.8)	13,797 (8.8)	33,815 (10.0)	42,333 (10.2)	14,855 (7.5)
	≥ 65 years, *n* (%)^a^	14,877 (7.1)	12,227 (7.9)	24,300 (7.2)	27,989 (6.7)	14,798 (7.5)
	Male, *n* (%)	105,308 (49.9)	78,546 (50.2)	169,960 (50.0)	208,819 (50.4)	102,210 (51.2)
	Concomitant drugs					
	Antipsychotics, *n* (%)^b^	15,969 (7.6)	14,698 (9.4)	25,920 (7.6)	30,887 (7.5)	21,453 (10.8)
	Antidepressants, *n* (%)^b^	39,814 (18.9)	35,492 (22.7)	61,562 (18.1)	72,161 (17.4)	48,816 (24.7)
Antipsychotics	Patiens, *n*	30,036	25,434	50,918	66,140	38,884
	Age, years					
	Mean (SD) ^a^	38.5 (15.4)	38.7 (15.2)	39.3 (15.6)	39.1 (16.1)	39.0 (15.8)
	≤ 19 years, *n* (%)^a^	3830 (12.8)	3211 (12.6)	6324 (12.4)	9189 (13.9)	5612 (14.4)
	≥ 65 years, *n* (%)^a^	1192 (4.0)	954 (3.8)	2233 (4.4)	2918 (4.4)	1232 (3.2)
	Male, *n* (%)	15,088 (50.2)	12,752 (50.1)	26,350 (51.7)	34,860 (52.7)	20,248 (52.1)
	Concomitant drugs					
	Anxiolytics, *n* (%)^b^	13,054 (43.5)	10,541 (41.4)	20,898 (41.0)	25,894 (39.2)	13,961 (35.9)
	Hypnotics, *n* (%)^b^	14,062 (46.8)	11,989 (47.1)	23,368 (45.9)	29,138 (44.1)	17,616 (45.3)
	Antidepressants, *n* (%)^b^	11,595 (38.6)	10,189 (40.1)	19,625 (38.5)	24,706 (37.4)	15,795 (40.6)
Antidepressants	Patiens, *n*	59,528	51,047	95,537	122,019	75,365
	Age, years					
	Mean (SD)^a^	41.0 (12.6)	41.9 (12.4)	41.6 (12.8)	42.0 (13.1)	43.7 (12.7)
	≤ 19 years, *n* (%)^a^	2906 (4.9)	2163 (4.2)	4508 (4.7)	6096 (5.0)	2901 (3.8)
	≥ 65 years, *n* (%)^a^	1688 (2.9)	1494 (2.9)	2791 (2.9)	3883 (3.2)	2409 (3.2)
	Male, *n* (%)	33,286 (55.9)	28,758 (56.3)	53,439 (55.9)	68,189 (55.9)	42,577 (56.5)
	Concomitant drugs					
	Anxiolytics, *n* (%)^b^	34,010 (57.1)	28,128 (55.1)	52,024 (54.5)	62,034 (50.8)	35,563 (47.2)
	Hypnotics, *n* (%)^b^	25,768 (43.3)	22,256 (43.6)	41,099 (43.0)	50,296 (41.2)	32,187 (42.7)
	Antipsychotics, *n* (%)^b^	9507 (16.0)	9210 (18.0)	16,169 (16.9)	19,808 (16.2)	14,422 (19.1)

^a^As of the initial month of prescription records for psychotropic drugs of each category in each segmented period

^b^Psychotropic drugs of other categories prescribed in the initial month of prescription records for drugs of each category in each segmented period

### Changes in the proportion of patients prescribed more than the standard number of drugs using segmented regression analysis

**Fig. 2:**
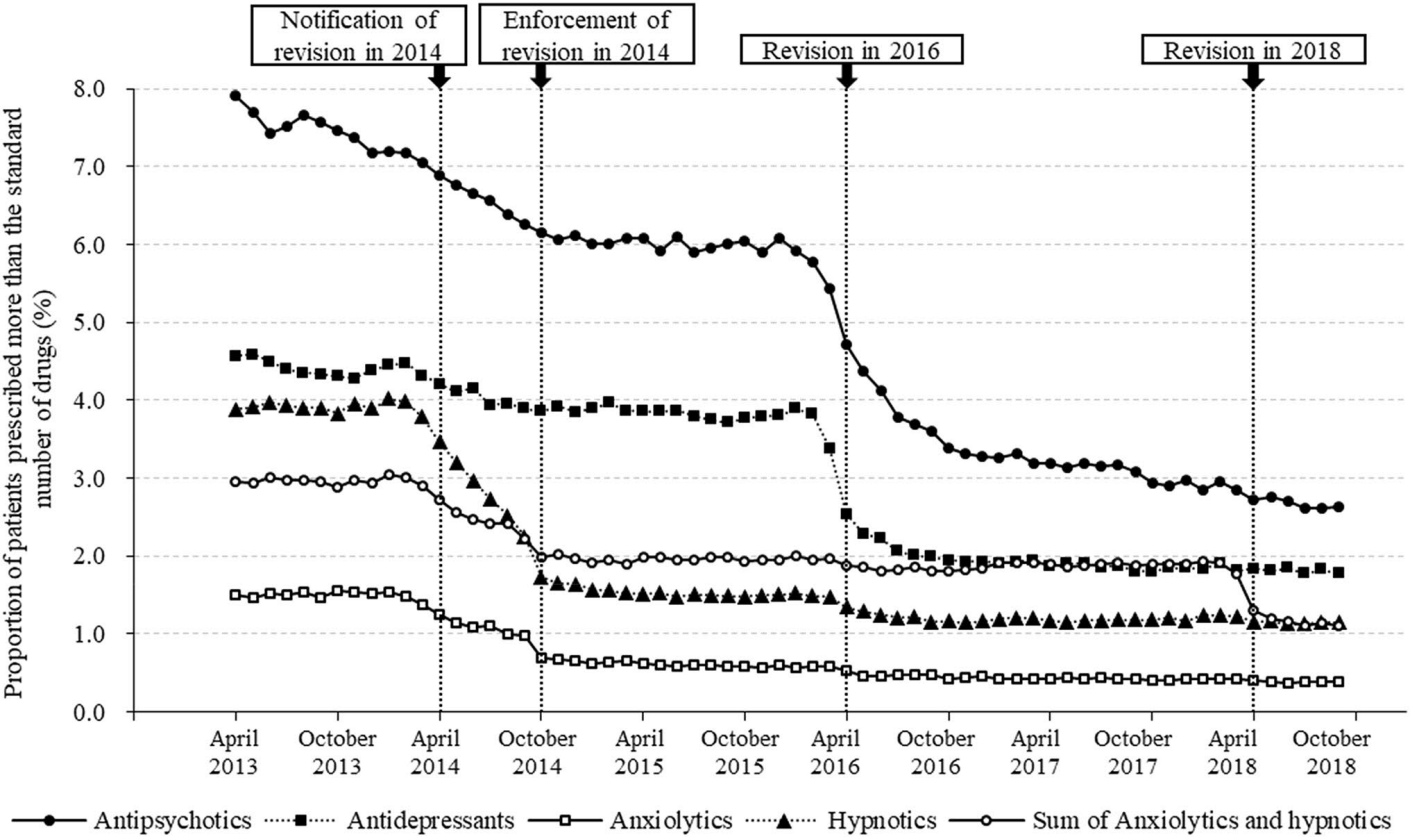
Trend of monthly proportions (%) of patients prescribed more than the standard number of drugs. The standard numbers are two for anxiolytics, hypnotics, antipsychotics, and antidepressants and three for the sum of anxiolytics and hypnotics

The trend of the monthly proportion (%) of patients prescribed more than standard number of drugs is shown in Fig. 2. Estimated changes in the proportion of patients prescribed more than the standard number of drugs are shown in Table 2.

**Table 2:** Estimated changes in the proportion (%) of patients prescribed more than standard number of drugs following revisions of medical service fees using segmented regression analysis

	Variable	Anxiolytics	Hypnotics	Sum of anxiolytics and hypnotics	Antipsychotics	Antidepressants
Baseline	Intercept (95% CI)	1.5226* (1.4889 to 1.5563)	3.9281* (3.8379 to 4.0183)	2.9606* (2.9112 to 3.0100)	7.9756* (7.3758 to 8.5754)	4.5121* (4.3939 to 4.6303)
	Trend (95% CI)	− 0.0037 (− 0.0082 to 0.0009)	− 0.0042 (− 0.0159 to 0.0074)	0.0002 (− 0.0065 to 0.0069)	− 0.0766* (− 0.1352 to − 0.018)	− 0.0152 (− 0.0313 to 0.0009)
Notification of the revision in 2014	Level change (95% CI)	− 0.2144* (− 0.2732 to − 0.1556)	− 0.1150* (− 0.2199 to − 0.0101)	− 0.1991* (− 0.2853 to − 0.1129)	− 0.0375 (− 0.3064 to 0.2314)	− 0.0623 (− 0.2695 to 0.1449)
	Trend change (95% CI)	− 0.0454* (− 0.0592 to − 0.0316)	− 0.2536* (− 0.2846 to − 0.2226)	− 0.0862* (− 0.1066 to − 0.0658)	− 0.0478 (− 0.1732 to 0.0776)	− 0.0493 (− 0.0979 to − 0.0007)
Enforcement of the revision in 2014	Level change (95% CI)	− 0.3043* (− 0.3521 to − 0.2565)	− 0.5509* (− 0.6418 to − 0.4600)	− 0.2858* (− 0.3560 to − 0.2156)	− 0.0561 (− 0.3064 to 0.1942)	0.0630 (− 0.1052 to 0.2312)
	Trend change (95% CI)	0.0431* (0.0298 to 0.0564)	0.2441* (0.2161 to 0.2721)	0.0859* (0.0664 to 0.1054)	0.0733 (− 0.0427 to 0.1893)	0.0507* (0.0039 to 0.0975)
Revision in 2016	Level change (95% CI)	− 0.0823* (− 0.1160 to − 0.0486)	− 0.1813* (− 0.2615 to − 0.1011)	− 0.1204* (− 0.1700 to − 0.0708)	− 0.6804* (− 0.9279 to − 0.4329)	− 1.5047* (− 1.6235 to − 1.3859)
	Trend change (95% CI)	0.0027 (− 0.0002 to 0.0057)	0.0115* (0.0027 to 0.0203)	0.0023 (− 0.0021 to 0.0066)	− 0.0305 (− 0.0924 to 0.0314)	− 0.0052 (− 0.0156 to 0.0052)
Revision in 2018	Level change (95% CI)	− 0.0049 (− 0.0602 to 0.0503)	− 0.0573 (− 0.1606 to 0.0460)	− 0.6045* (− 0.6854 to − 0.5236)	− 0.0854 (− 0.3543 to 0.1835)	0.1045 (− 0.0899 to 0.2989)
	Trend change (95% CI)	0.0012 (− 0.0119 to 0.0144)	0.0058 (− 0.0218 to 0.0335)	− 0.0358* (− 0.0551 to − 0.0165)	0.0736 (− 0.0375 to 0.1847)	0.0096 (− 0.0367 to 0.0559)

Time unit of baseline trend and trend change is per month

*p < 0.05

After notification of the revision in 2014, there were significant decreases in the levels and trends for anxiolytics, hypnotics, and the sum of anxiolytics and hypnotics. For these drug categories, after enforcement of the revision in 2014, the levels decreased further, while the trends increased almost to the baseline trend for these drug categories. However, there were no significant changes in the levels or trends for antipsychotics and antidepressants after the notification and enforcement of the revision in 2014.

After the revision in 2016, there were significant decreases in the levels for antipsychotics and antidepressants. There was additionally a significant decrease in the levels for anxiolytics, hypnotics, and the sum of anxiolytics and hypnotics and a slight increase in the trend for hypnotics.

After the revision in 2018, there were significant decreases in the levels and trends only for the sum of anxiolytics and hypnotics. There were no significant changes in the level or trend for other drug categories.

### Changes in the proportions of patients exhibiting a decrease in number of prescribed drugs from more than the standard to within the standard

**Fig. 3:**
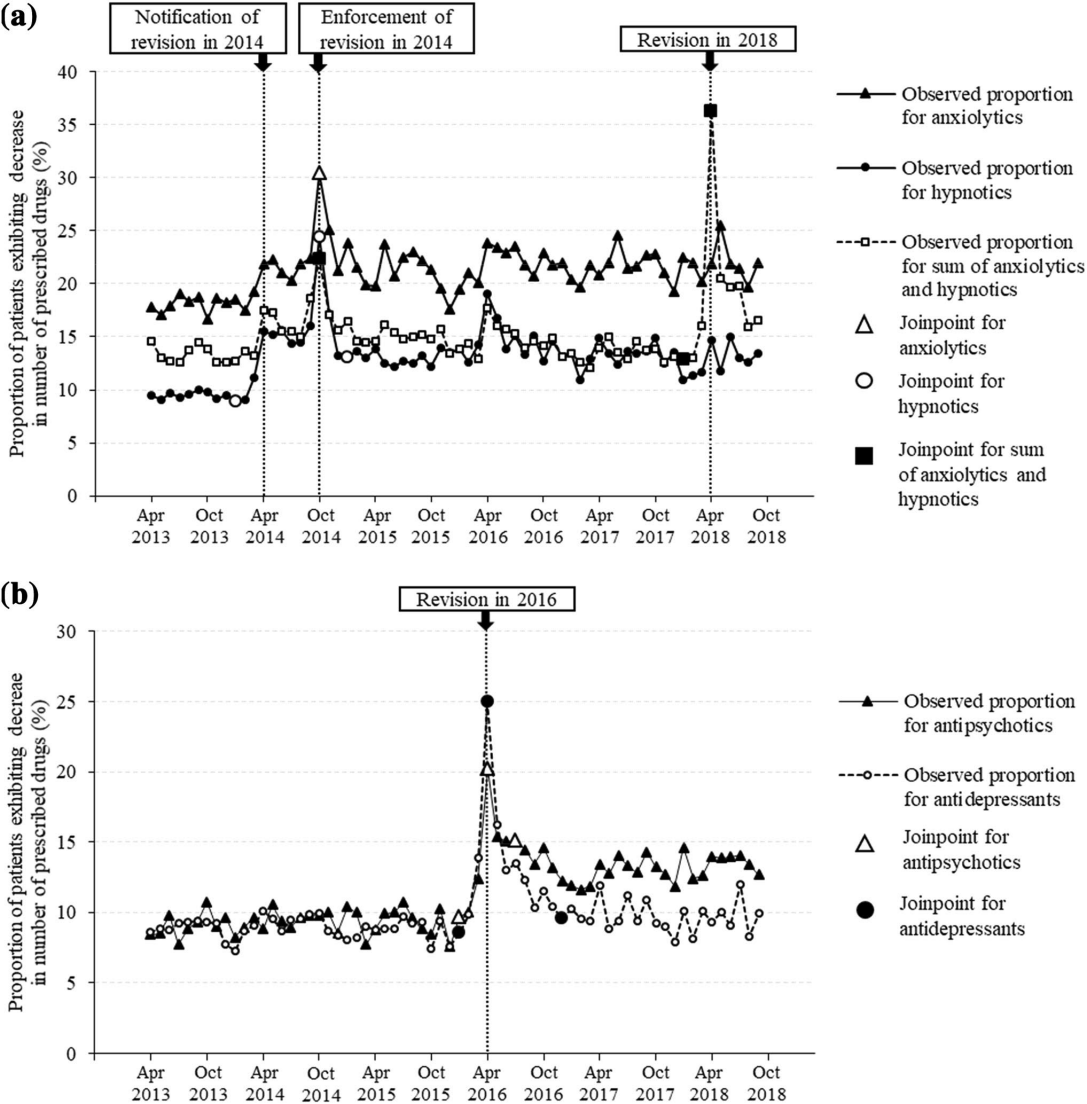
Trend of monthly proportions (%) of patients exhibiting a decrease in the number of prescribed drugs from more than the standard number to within the standard number and joinpoints identified using joinpoint regression analysis: **a** anxiolytics, hypnotics, and sum of anxiolytics and hypnotics, **b** antipsychotics and antidepressants. The standard numbers are two for anxiolytics, hypnotics, antipsychotics, and antidepressants and three for the sum of anxiolytics and hypnotics

Figure 3 presents the trend of monthly proportions (%) of patients exhibiting a decrease in the number of prescribed drugs from more than the standard number to within the standard number; joinpoints were identified using joinpoint regression analysis. Regression lines and estimated slopes are shown in Supplementary Figure S1 and Supplementary Table S2, respectively.

For anxiolytics, one joinpoint in October 2014 was identified. The identified joinpoint coincided with the enforcement of the revision in 2014. The best fitting joinpoint regression model indicated that the proportion increased at the first segment and subsequently stabilized at the second segment. The observed proportion in October 2014 was 30.4%, while all other proportions were 16.7–25.5%.

For hypnotics, three joinpoints were identified in January 2014, October 2014, and January 2015. The second joinpoint in October 2014 coincided with the enforcement of the revision in 2014, and notification of the revision in 2014 was included in the second segment. The best fitting model indicated that the proportion increased in the second segment and decreased in the third segment. The observed proportion in April 2014 was 15.5%, while the proportions in the first segment were 9.1–10.0%. The observed proportion in October 2014 was 24.4%, while other proportions were 19.1% in April 2016 or less (most values were less than 16.0%).

For the sum of anxiolytics and hypnotics, three joinpoints were identified in October 2014, January 2018, and April 2018. The third joinpoint coincided with the revision in 2018. The best fitting model indicated that the proportion increased in the third segment and decreased in the fourth segment. The observed proportion in April 2018 was 36.3%, while all other proportions were 12.1–22.3%. In the subgroup analysis, the observed proportions with a decrease in anxiolytics, hypnotics, and both anxiolytics and hypnotics in April 2018 were 23.1%, 9.7%, and 3.5%, respectively (Supplementary Figure S2).

For antipsychotics, three joinpoints were identified in January 2016, April 2016, and December 2016. The second joinpoint in April 2016 coincided with the revision in 2016. The best fitting model indicated that the proportion increased in the second segment and decreased in the third segment. The observed proportion in April 2016 was 20.2%, while all other proportions ranged from 7.6% to 15.4%.

For antidepressants, three joinpoints were identified in January 2016, April 2016, and July 2016. The second joinpoint in April 2016 coincided with the revision in 2016. The best fitting model indicated that the proportion increased in the second segment and decreased in the third segment. The observed proportion in April 2016 was 25.0%, while all other proportions were 7.3–16.3%.

Results of the sensitivity analysis showed the same tendency as the results of the main analysis, while overall proportions in the sensitivity analysis were higher than that in the main analysis (data not shown).

## Discussion

Using a large Japanese claims database, we investigated the effect of the revision in 2018 on the proportion of patients prescribed more than the standard number of drugs and the effect of the revisions in 2014, 2016, and 2018 on changes in drug prescription at an individual patient level, especially a decrease in the number of drugs. The proportion of patients prescribed more than the standard number of drugs for the sum of anxiolytics and hypnotics abruptly decreased after the revision in 2018, while no significant changes were identified for other drug categories. In addition, the proportion of anxiolytics and hypnotics decreased after the revision in 2014, and the proportion of antipsychotics and antidepressants decreased after the revision in 2016. For all drug categories, the proportion of patients exhibiting a decrease in the number of prescribed drugs from more than the standard number to within the standard number were temporally higher when the revisions were enforced than that at other timepoints.

A previous study reported the association of the revisions in 2014 and 2016 with a decrease in the proportion of patients prescribed three or more drugs for anxiolytics, hypnotics, antipsychotics, and antidepressants [17]. Our study results were generally consistent with this finding. In addition, our study showed that the revision in 2014 was associated with a decrease in the proportion of patients prescribed four or more drugs for the sum of anxiolytics and hypnotics, while a previous study reported no significant changes in the proportion of patients prescribed three or more drugs after the revision in 2014 [17]. This inconsistency was most likely due to differences in the outcome definitions. It is not surprising that the proportion of patients prescribed four or more drugs was more likely to decrease than that of patients prescribed three or more drugs, because the standard number according to the revision in 2014 was two.

Our study showed that the proportion of patients exhibiting a decrease in the number of drugs from more than the standard number to within the standard increased when the revisions were enforced (in October 2014 for anxiolytics and hypnotics, in April 2016 for antipsychotics and antidepressants, and in April 2018 for the sum of anxiolytics and hypnotics). Therefore, our study suggested that these revisions promoted a reduction in the number of drugs prescribed, which played an important role in decreasing the proportion of patients prescribed more than the standard number of drugs.

According to the revision in 2018, the simultaneous prescription of two anxiolytics and two hypnotics (four drugs in total) was subject to reduction of medical service fees. Our study suggested that the revision in 2018 succeeded in reducing those prescriptions, because there was a significant decrease in the proportion of patients prescribed more than the standard number of drugs (four or more drugs) for sum of anxiolytics and hypnotics, while there were no significant changes in the proportion of patients prescribed more than the standard number of drugs (three or more drugs) for anxiolytics or hypnotics. In addition, subgroup analysis showed that among the patients exhibiting a decrease in the number of prescribed drugs, the highest proportion of patients exhibiting a reduction in only anxiolytic prescriptions formed the highest proportion in April 2018, while those exhibiting a reduction in only hypnotic prescriptions formed the highest proportion in October 2014. Thus, it is possible that prescription patterns and priority of drugs to be reduced varied depending on the period.

Several limitations of our study need to be considered. First, our results might be biased if there were other events affecting the outcomes around the time of each revision. However, as far as we know, there were no other important events around the time of each revision. Second, our definition of a decrease in the number of prescribed drugs could not accurately distinguish the actual decrease from the decrease associated with switching drugs. Thus, the absolute value of these proportions might overestimate the actual proportions; however, we believe that our study could appropriately evaluate changes in the trend of decrease, because we considered these relative changes. Third, the database did not cover patients aged 75 or more. In addition, beneficiaries in the database may be wealthier than the general population and psychiatric disease of patients in the database may be less severe than that of patients in the general population, because beneficiaries in the database were employed workers and their families. Thus, the generalizability of our results may be limited. Fourth, our study design could not evaluate the effect of the revision in 2018 on long-term use of anxiolytics and hypnotics. Further study needs to evaluate data over a sufficiently long period. Fifth, our study design could not evaluate the effectiveness of drugs with decreased prescriptions. Reducing psychotropic drugs is not always appropriate [32] and establishing further evidence is important for stopping irrational psychotropic polypharmacy, especially evidence regarding when and how to reduce drugs.

## Conclusion

Our study suggested that the 2018 revision of medical service fees aimed at reducing irrational psychotropic polypharmacy led to a decrease in the proportion of patients prescribed more than the standard number of drugs for the sum of anxiolytics and hypnotics. In addition, the revisions of medical service fees in 2014, 2016, and 2018 promoted a reduction in the number of prescribed drugs, which was an important factor contributing to the decrease in the proportion of patients prescribed more than the standard number of drugs for all drug categories. Further study is needed to evaluate the effect of the revision in 2018 on long-term use of anxiolytics and hypnotics. In addition, establishing further clinical evidence that is helpful for reducing psychotropic drugs is needed.

## Supplementary Information

Below is the link to the electronic supplementary material.

Supplementary file1 (PDF 210 KB)

## Data Availability

The data that support the findings of this study are available from JMDC Co. Ltd (Tokyo, Japan); however, restrictions apply to the availability of these data, which were used under license for the current study, and thus the data are not publicly available. The data are, however, available from the authors upon reasonable request and with permission from JMDC Co. Ltd (Tokyo, Japan).
