# Supplementary material for: Effects of medical service fee revision on reducing irrational psychotropic polypharmacy in Japan: an interrupted time-series analysis
Source: Soc Psychiatry Psychiatr Epidemiol. 2021 Jul 31;57(2):411–22. doi: 10.1007/s00127-021-02147-0 (PMC8784362; doi:10.1007/s00127-021-02147-0)
Supplement: Supplementary file 1 — Supplementary file1 (PDF 210 KB) [file 127_2021_2147_MOESM1_ESM.pdf]

Supplementary file

Effects of medical service fee revision on reducing irrational psychotropic polypharmacy in Japan: an interrupted time series analysis

Yusuke Okada [1], Manabu Akazawa [1]

1 Department of Public Health and Epidemiology, Meiji Pharmaceutical University, Tokyo, Japan

*Correspondence should be addressed to: Manabu Akazawa, Ph.D, MPH

E-mail: makazawa@my-pharm.ac.jp

Department of Public Health and Epidemiology, Meiji Pharmaceutical University, 2-522-1, Noshio, Kiyose, Tokyo, 204-8588, Japan

Supplementary Table S1. List of psychotropic drugs

Category	Generic name
Anxiolytics	Alprazolam
	Bromazepam
	Chlordiazepoxide
	Clorazepate dipotassium
	Clotiazepam
	Cloxazolam
	Diazepam
	Ethyl loflazepate
	Etizolam
	Fludiazepam
	Flutazolam
	Flutoprazepam
	Gamma oryzanol
	Hydroxyzine hydrochloride
	Hydroxyzine pamoate
	Lorazepam
	Medazepam
	Mexazolam
	Oxazolam
	Tandospirone citrate
	Tofisopam
Hypnotics	Amobarbital
	Barbital
	Bromovalerylurea
	Brotizolam
	Chloral hydrate
	Chlorpromazine hydrochloride/ promethazine hydrochloride/ phenobarbital
	Estazolam
	Eszopiclone
	Flunitrazepam
	Flurazepam hydrochloride
	Haloxazolam
	Lormetazepam
	Nimetazepam
	Nitrazepam
	Pentobarbital calcium

	Phenobarbital
	Phenobarbital sodium
	Quazepam
	Ramelteon
	Rilmazafone hydrochloride hydrate
	Suvorexant
	Triazolam
	Triclofos sodium
	Zolpidem tartrat
	Zopiclone
Antipsychotics	Aripiprazole
	Asenapine maleate
	Blonanserin
	Bromperidol
	Carpipramine hydrochloride hydrate
	Carpipramine maleate
	Chlorpromazine hydrochloride
	Chlorpromazine phenolphthalinate
	Clocapramine hydrochloride hydrate
	Clozapine
	Fluphenazine maleate
	Fluphenazine decanoate
	Haloperidol
	Haloperidol decanoate
	Levomepromazine maleate
	Mosapramine hydrochloride
	Moperone hydrochloride
	Nemonapride
	Olanzapine
	Oxypertine
	Paliperidone
	Paliperidone palmitate
	Perospirone hydrochloride hydrate
	Perphenazine
	Perphenazine fendizoate
	Perphenazine maleate
	Pimozide
	Pipamperone hydrochloride

	Prochlorperazine maleate
	Propericiazine
	Quetiapine fumarate
	Reserpine
	Risperidone
	Spiperone
	Sulpiride
	Sultopride hydrochloride
	Timiperone
	Trifluoperazine maleate
	Zotepine
Antidepressants	Amitriptyline hydrochloride
	Amoxapine
	Clomipramine hydrochloride
	Dosulepin hydrochloride
	Duloxetine hydrochloride
	Escitalopram oxalate
	Fluvoxamine maleate
	Imipramine hydrochloride
	Lofepramine hydrochloride
	Maprotiline hydrochloride
	Mianserin hydrochloride
	Milnacipran hydrochloride
	Mirtazapine
	Nortriptyline hydrochloride
	Paroxetine hydrochloride hydrate
	Pemoline
	Sertraline hydrochloride
	Setiptiline maleate
	Trazodone hydrochloride
	Trimipramine maleat
	Venlafaxine hydrochloride

Supplementary Table S2. Results of joinpoint regression analysis

drug category	Segment	Period	Slope estimate (95% CI)
Anxiolytics	1	April 2013 to September 2014	0.3083 (0.1727 to 0.4439)
	2	October 2014* to September 2018	−0.0193 (−0.0514 to 0.0128)
Hypnotics	1	April 2013 to December 2013	−0.0172 (−0.3774 to 0.343)
	2	January 2014* to September 2014	1.1305 (0.6999 to 1.5611)
	3	October 2014* to December 2014	−1.979 (−5.9251 to 1.9671)
	4	January 2015* to September 2018	−0.0082 (−0.0413 to 0.0249)
Sum of anxiolytics and hypnotics	1	April 2013 to September 2014	0.2372 (0.1339 to 0.3405)
	2	October 2014* to December 2017	−0.1095 (−0.1432 to −0.0758)
	3	January 2018* to March 2018	4.7607 (1.5449 to 7.9765)
	4	April 2018* to September 2018	−2.4452 (−3.1643 to −1.7261)
Antipsychotics	1	April 2013 to December 2015	0.0016 (−0.028 to 0.0312)
	2	January 2016* to March 2016	2.5197 (0.2271 to 4.8123)
	3	April 2016* to November 2016	−0.5502 (−0.8565 to −0.2439)
	4	December 2016* to September 2018	0.0615 (0.0031 to 0.1199)
Antidepressants	1	April 2013 to December 2015	−0.0249 (−0.0576 to 0.0078)
	2	January 2016* to March 2016	4.1527 (1.6278 to 6.6776)
	3	April 2016* to June 2016	−3.3201 (−5.845 to −0.7952)
	4	July 2016* to September 2018	−0.0689 (−0.1155 to −0.0223)

* Identified joinpoint

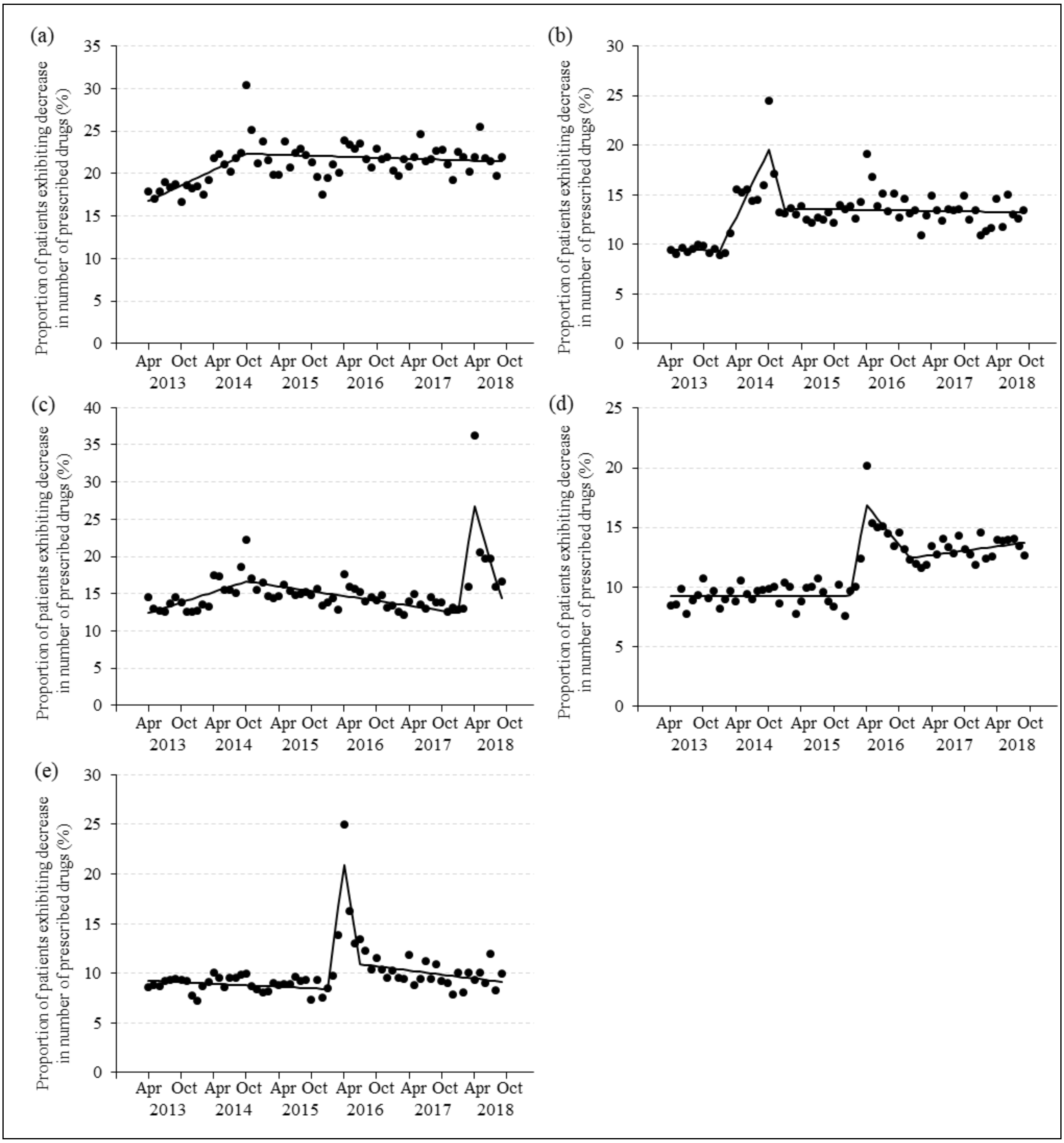

Supplementary Figure S1. Trend of monthly proportions of patients exhibiting a decrease in the number of prescribed drugs from more than the standard number to within the standard number and regression lines using joinpoint regression analysis: (a) anxiolytics, (b) hypnotics, (c) sum of anxiolytics and hypnotics, (d) antipsychotics, (e) antidepressants. The standard numbers are two for anxiolytics, hypnotics, antipsychotics, and antidepressants, and three for the sum of anxiolytics and hypnotics. Circle, observed proportion; solid line, estimated regression line

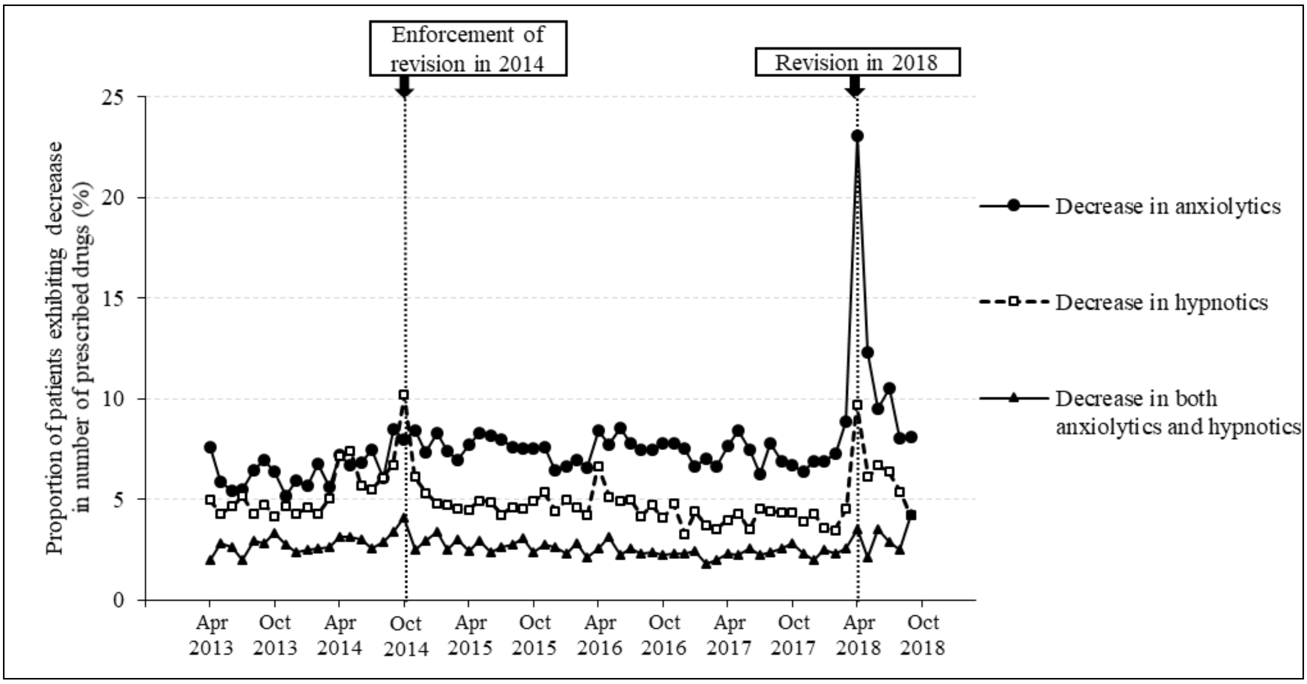

Supplementary Figure S2. Trend of monthly proportions of patients exhibiting a decrease in the number of prescribed drugs for sum of anxiolytics and hypnotics from four or more to three or less by the type of drugs that decreased
